# Comparison of Sizing Methods to Optimize Device Selection for Percutaneous Closure of Atrial Septal Defects

**DOI:** 10.1155/crp/1663610

**Published:** 2026-09-30

**Authors:** Çağdaş Vural, Ali Baykan, Alper Doğan, Yunus Emre Kum, Özge Pamukçu Akay, Kazım Üzüm, Nazmi Narin

**Affiliations:** ^1^ Pediatric Cardiology Department Medical Faculty Mustafa Eraslan Children Hospital Erciyes University Melikgazi Kayseri 38030 Turkey erciyes.edu.tr; ^2^ Eskişehir City Hospital, 71 Evler Neighborhood Çavdarlar Street Odunpazarı, Eskişehir 26080, Turkey; ^3^ Pediatric Cardiology Department Aksaray University Training and Research Hospital Aksaray 68000 Turkey; ^4^ Pediatric Cardiology Department Medical Faculty Marmara University Pendik Training and Research Hospital Marmara University Pendik İstanbul Turkey marmara.edu.tr; ^5^ Pediatric Cardiology Department, Acıbadem Hospital- KAYSERİ, Mustafa Kemal Paşa Blv. No:1 Seyitgazi Melikgazi, Kayseri 38030, Turkey; ^6^ Pediatric Cardiology Department Medical Faculty İzmir Kâtip Çelebi University Çiğli İzmir 35620 Turkey

**Keywords:** atrial septal defect (ASD), congenital heart diseases, interventional cardiology, sizing methods, transcatheter closure

## Abstract

**Background and Aims:**

Accurate sizing of atrial septal defects (ASDs) is crucial for appropriate device selection during percutaneous transcatheter closure. Despite the availability of multiple imaging and sizing techniques, no consensus exists on the optimal method for determining device size. This study aimed to compare preprocedural ASD measurements obtained using different techniques with the final device waist diameter and to identify the measurement method demonstrating the most favorable agreement and reproducibility.

**Methods:**

This retrospective study included 149 pediatric patients (64 males, 85 females) who underwent percutaneous transcatheter ASD closure. Preprocedural defect sizing was performed using transthoracic echocardiography (TTE), balloon sizing, and transesophageal echocardiography (TEE). TEE measurements were obtained in the four‐chamber, aortic, and bicaval views. In addition to individual diameters, the arithmetic mean, cube root–derived diameter, and maximal TEE‐measured diameter were calculated and compared with the final device waist diameter. The final device waist diameter, measured fluoroscopically after deployment, was used as the reference comparator, reflecting operator‐selected device sizing rather than true anatomical defect dimensions.

**Results:**

Among TEE‐based measurements, both the cube root–derived diameter and the arithmetic mean of TEE measurements demonstrated high reproducibility and favorable agreement with the final device waist diameter, with only marginal numerical differences between the two methods. Bland–Altman analysis supported these findings by showing minimal bias and narrow limits of agreement, whereas TTE and balloon sizing demonstrated lower, though statistically significant, concordance with the final device waist diameter.

**Conclusion:**

Combining multiple measurement techniques improves agreement with the operator‐selected device size during percutaneous ASD closure. While TTE alone may be sufficient in patients with simple defects and adequate acoustic windows, integrated approaches incorporating TEE and multiaxis measurements showed favorable agreement characteristics, particularly in anatomically heterogeneous defects or resource‐limited settings. These findings should be interpreted as reflecting agreement with operator‐selected device sizing rather than anatomical sizing accuracy, given the inherent circularity of using implanted device size as a reference standard.

## 1. Introduction

Atrial septal defect (ASD) is one of the most common congenital heart diseases with an incidence of 1.6 per 1000 live births [1]. Asymptomatic infants with small ASDs are followed, as spontaneous closure is expected. Those that do not close spontaneously or have clinical significance require closure. Percutaneous occlusion should be performed in cases with favorable anatomy, hemodynamic repercussions, or a history of paradoxical embolism [2].

Successful nonsurgical closure of ASD was first reported in 1974 by King and Mills [3]. Since then, technical advances have been made in the transcatheter closure method. The location and size of the ASD and the feasibility of transcatheter closure can be determined by various imaging techniques to minimize complications and achieve effective closure with an appropriately sized device. Over time, with increasing experience in transcatheter closure, various methods have been used to determine the optimal device size.

A device that is too large can lead to compression of surrounding cardiovascular structures and serious complications such as cardiac erosion. Conversely, a device that is too small can increase the risk of instability, distal embolization, and residual shunting, making it an inappropriate choice. Accurate sizing of the defect is critically important for selecting the appropriate device size [4]. Therefore, accurate determination of the anatomy and size of the ASD is crucial to reducing complications and achieving procedural success [5]. However, there has been a lack of consensus on how to choose the ideal device size.

In this study, we aimed to compare the ASD sizes measured by different methods (transthoracic echocardiography [TTE], transesophageal echocardiography [TEE], and balloon sizing during transcatheter closure) with the device waist diameter measured after percutaneous ASD closure in children. Additionally, we aimed to identify the measurement method with the closest concordance to the final device waist diameter and to share our institutional experience regarding the success, efficacy, and safety of percutaneous closure of ASD.

## 2. Materials and Methods

This retrospective study evaluated the medical records of pediatric patients who underwent percutaneous ASD closure. Demographic data included age, sex, weight, preprocedural symptoms, and comorbidities, including associated cardiac defects and noncardiac diseases. Angiographic and procedural data comprised balloon sizing diameter, mean pulmonary artery pressure, procedure and fluoroscopy times, radiation dose, and ASD diameters measured by TTE and TEE. Device‐related data, including the type and nominal size of the transcatheter closure devices, were also recorded. In addition, procedure‐related complications and concomitant interventional procedures were documented.

### 2.1. Exclusion Criteria

The exclusion criteria included patients older than 18 years, those with multiple cribriform ASDs, and cases with incomplete measurement data. Patients with additional small, hemodynamically insignificant defects were not excluded; however, these defects were not included in diameter calculations. Because cribriform occluder devices have relatively small waist diameters despite larger disc coverage areas, patients treated with cribriform devices were excluded from device size comparison analyses to avoid potential distortion of size‐based comparisons.

To preserve methodological consistency and minimize selection bias, only patients who underwent all measurement techniques during the same procedure were included. Although this approach may limit the generalizability of the findings, it allowed for a valid and consistent statistical comparison among the measurement techniques.

### 2.2. Evaluation With TEE and/or TTE

TTE was performed in all patients as part of the initial diagnostic evaluation and decision‐making process for ASD closure. The ASD diameter was measured primarily in the subcostal view. However, TTE measurements were not used alone and were interpreted together with other measurement modalities for final device selection. TTE was also preferred in patients with an adequate acoustic window that allowed sufficient visualization of the defect.

Echocardiographic measurements were performed by experienced pediatric cardiologists/echocardiographers. Each case was independently assessed by more than one operator, and the final measurements used for analysis were established by consensus under senior supervision, reflecting routine clinical practice at our institution.

Preprocedural TTE examinations were performed by a pediatric cardiologist using the GE Vivid‐7 Pro (General Electric Healthcare, USA) and Philips Affiniti 50C (Philips Healthcare, Andover, Netherlands) systems equipped with 3–7 MHz transducers for two‐dimensional (2D) imaging and color Doppler flow mapping.

Before and during the procedure, all patients underwent comprehensive TEE evaluation to assess the morphology, location, number, and size of the defects, as well as rim adequacy and the presence of associated congenital heart anomalies. TEE was particularly preferred in patients with large or multiple defects, deficient rims, or additional complex cardiac anomalies.

Using TEE, the defect diameter was measured in multiple planes to determine the maximal defect size. The most informative views for defect sizing were the mid‐esophageal four‐chamber view at 0°, the short‐axis view at 45°–60°, and the bicaval long‐axis view at 90°–110°. In addition to the maximal defect diameter and rim measurements, the arithmetic mean of the measured diameters and the cube root of the product of all defect diameters obtained from different TEE planes were also calculated.

### 2.3. Procedure for ASD Closure

Written informed consent was obtained from the parents of all patients. All procedures were performed under general anesthesia and guided by fluoroscopy, TEE, and TTE. Prophylactic ampicillin (50 mg/kg) was administered before the procedure, and heparin (100 IU/kg) was administered during the procedure.

The pulmonary‐to‐systemic blood flow ratio (Qp/Qs) was calculated in the catheterization laboratory.

Vascular access was generally obtained through the right femoral vein. A multipurpose catheter was advanced into the right atrium and subsequently into the left atrium through the ASD. The catheter was positioned in the left upper pulmonary vein using an exchange guidewire. A deflated sizing balloon catheter was then advanced over the guidewire into the left atrium and inflated with a mixture of contrast agent and saline solution. Stop‐flow balloon sizing was performed under echocardiographic guidance, and the stretched ASD diameter was measured fluoroscopically.

The type and size of the ASD occluder device were selected according to TEE findings and balloon sizing measurements. The delivery sheath was advanced through the right femoral vein over a long exchange guidewire. Device deployment was performed only after echocardiographic confirmation of appropriate positioning without significant residual shunting, atrioventricular valve dysfunction, or venous obstruction.

Successful implantation was defined as stable device deployment in the intended position without malposition or embolization in the catheterization laboratory. All ASD closure procedures were performed by the same experienced operators throughout the study period.

### 2.4. Postprocedural Assessment and Follow‐Up

After the procedure, the widest device waist diameter was measured fluoroscopically in the 30° left anterior oblique/30° cranial projection using the standardized caliper software integrated into the fluoroscopy system. Measurements were performed in the presence of the entire procedural team, and the final value was recorded upon consensus agreement among all observers.

All obtained measurements were compared with the final device waist diameter. These measurements included TEE‐derived defect diameters obtained from the four‐chamber, aortic short‐axis, and bicaval views; the arithmetic mean of all TEE measurements; the cube root of the product of all TEE‐derived diameters; the defect diameter measured by balloon sizing during the procedure; and the defect diameter measured by TTE before the intervention.

Physical examination, electrocardiography (ECG), and TTE were performed in all patients immediately after the procedure, at 24 h, and at the 1st, 3rd, and 6th months of follow‐up. All patients routinely received acetylsalicylic acid therapy (3–5 mg/kg/day) for 6 months after the procedure.

Although residual confounding is an inherent limitation of retrospective observational studies, all patients were managed at a single center by the same clinical team using largely standardized periprocedural protocols, which likely reduced practice‐related variability and potential bias.

This study was approved by the Erciyes University Clinical Research Ethics Committee (Decision No.: 2018/310, June 8, 2018). Written informed consent was obtained from the parents of all patients.

### 2.5. Statistical Analysis

Statistical analyses were performed using IBM SPSS Statistics for Windows, Version 22.0 (IBM Corp., Armonk, NY, USA). Continuous variables were expressed as mean ± standard deviation (SD).

Agreement between different ASD diameter measurement techniques and the final device waist diameter was assessed using Bland–Altman analysis and intraclass correlation coefficients (ICCs).

For the Bland–Altman analysis, the difference between each measurement technique and the device waist diameter was calculated for each patient. The mean difference (bias) and 95% limits of agreement (LoA) were determined as the mean difference ± 1.96 SD. Bland–Altman plots were generated by plotting the differences against the mean of the two measurements to evaluate systematic bias and the degree of agreement between methods.

Reliability and agreement between each measurement technique and the final device waist diameter were further evaluated using ICC analysis. ICC was selected as the primary reliability metric because it accounts for both correlation and agreement simultaneously, making it particularly appropriate for method comparison studies involving continuous measurements. Unlike Pearson’s correlation coefficient, which captures only the direction and strength of linear association without accounting for systematic bias, ICC incorporates both systematic and random error components. Weighted kappa, while suitable for ordinal data, is less appropriate for continuous clinical measurements such as defect diameters and does not provide estimates of measurement error magnitude. ICC values were calculated separately for each measurement modality using a two‐way mixed‐effects model with absolute agreement and single‐measurement units, as recommended for method comparison studies.

Categorical variables were presented as frequencies and percentages and compared using the chi‐square test or Fisher’s exact test, as appropriate. The association between rim deficiency and the presence of complications was specifically evaluated using the chi‐square test.

All statistical tests were two‐tailed, and a p value < 0.05 was considered statistically significant.

## 3. Results

A total of 346 patients who underwent percutaneous ASD closure between June 2016 and December 2025 were screened. Patients older than 18 years (*n* = 4), those with cribriform ASD (*n* = 19), and those with incomplete datasets for all measurement modalities (*n* = 174) were excluded (Figure 1).

**FIGURE 1:**
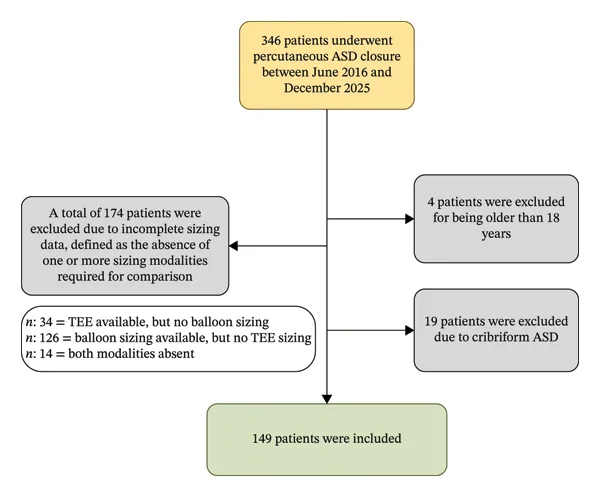
Flow diagram of patient selection and application of inclusion and exclusion criteria.

The final study population consisted of 149 children (64 boys and 85 girls) aged between 15 months and 17 years. The median age was 5.5 years (IQR: 3–11 years), and the mean body weight was 27.7 ± 19.5 kg.

The mean defect diameter measured by TTE was 14.0 ± 5.5 mm. The mean defect diameter obtained by balloon sizing during angiographic evaluation was 15.6 ± 5.2 mm. Defect diameters measured by TEE in the four‐chamber, aortic short‐axis, and bicaval long‐axis views were 13.1 ± 4.6 mm, 13.2 ± 4.9 mm, and 13.4 ± 4.9 mm, respectively.

The arithmetic mean of all TEE‐derived defect diameters was 13.2 ± 4.6 mm. Similarly, the mean cube root of the product of all TEE‐derived diameters was 13.2 ± 4.6 mm, whereas the mean maximal defect diameter measured by TEE was 14.6 ± 5.0 mm. The mean postdeployment device waist diameter measured fluoroscopically was 13.4 ± 4.9 mm (Table 1).

**TABLE 1:** Demographic and angiographic data of patients and defect diameters obtained by various techniques.

Patient who underwent cardiac catheterization (*n*:149)	64 boys (43%), 85 girls (57%)
Patient age (years)	5.5 (3‐11)^a^
Patient body weight (kg)	27.7 ± 19.5 kg^b^
Defect size (measured by transthoracic echocardiography) (n:149)	14.0 ± 5.5 mm^b^
Defect size measured by balloon sizing technique (n:149)	15.6 ± 5.2 mm^b^
Defect sizes measured by TEE (n:149)	
Four‐chamber axis	13.1 ± 4.6 mm^b^
Aortic axis	13.2 ± 4.9 mm^b^
Bicaval axis	13.4 ± 4.9 mm^b^
Arithmetic mean of all axis defect diameters	13.2 ± 4.6 mm^b^
The cube root of the product of all defect diameters	13.2 ± 4.6 mm^b^
The largest diameter of all TEE axis diameters	14.6 ± 5.0 mm^b^
Device size	16.4 ± 5.7 mm^b^
Device waist diameter	13.4 ± 4.9 mm^b^
Device types:	
Amplatzer Septal Occluder	105 (∼70.5%)
CeraFlex Septal Occluder	32 (∼21.5%)
Occlutech Atrial Septal Occluder	8 (∼5.4%)
MemoPart Septal Occluder	4 (∼2.7%)
Additional cardiac anomalies:	
Mitral valve prolapse + mitral regurgitation	13
Pulmonary stenosis	11
Bicuspid aortic valve with aortic stenosis	3
Persistent left superior vena cava	1
Ebstein anomaly	1
Small PDA and small VSD	1
Interruption of VCI with azygos vein continuation	1
Mild pulmonary hypertension	1
Other interventions during the ASD closure procedure	
Pulmonary balloon valvuloplasty	3
Aortic balloon valvuloplasty	1
Balloon‐assisted technique	9

^a^Median (IQR).

^b^(Mean ± SD).

Device size refers to the nominal manufacturer‐labeled size, whereas device waist diameter refers to the measured diameter after deployment.

The devices used in the study were as follows: Amplatzer Septal Occluder (Abbott Medical, St. Paul, MN, USA) in 105 patients (70.5%), Occlutech Septal Occluder (Occlutech International, Helsingborg, Sweden) in 8 patients (5.4%), MemoPart Septal Occluder (LEPU Medical Technology Company, China) in 4 patients (2.7%), and CeraFlex Septal Occluder (Lifetech, Shenzhen, China) in 32 patients (21.5%).

Associated cardiac anomalies were present in 32 patients. Mitral valve prolapse with mitral regurgitation was identified in 13 patients, pulmonary valve stenosis in 11 patients, and bicuspid aortic valve with aortic stenosis in 3 patients. One patient had a small patent ductus arteriosus (PDA) and a small ventricular septal defect (VSD), one had Ebstein anomaly, one had interruption of the inferior vena cava with azygos continuation, one had a persistent left superior vena cava, and one had mild pulmonary hypertension.

In the patient with mild pulmonary hypertension, transcatheter closure was successfully performed after a balloon occlusion test confirmed hemodynamic and anatomical suitability (Table 1).

Patients with multiple ASDs requiring a large single device to cover both defects were not included in the study.

### 3.1. Major and Minor Complications

Shrivastava et al. [6] defined the major complications associated with transcatheter ASD closure as death, cerebral embolism, cardiac perforation with tamponade, endocarditis, repeat surgery, cardiac arrhythmias requiring permanent pacemaker implantation or long‐term antiarrhythmic therapy, and device embolization requiring urgent surgical removal.

In our study, all ASDs were successfully closed. None of the major complications described above—including death, cardiac perforation with tamponade, endocarditis, or repeat surgery—were observed during the procedure or follow‐up period.

Overall, complications occurred in 20 patients. Arrhythmia was the most common complication and was observed in 15 patients (transient or permanent), followed by thromboembolic events in 2 patients and device migration in 3 patients.

Among patients with arrhythmia, 14 experienced transient events that resolved spontaneously or after a single pharmacological intervention without recurrence. Two patients received corticosteroid therapy for ventricular and supraventricular ectopic beats (VEs and SVEs), one patient was treated with lidocaine for ventricular ectopic beats, and atropine was administered to two patients because of atrioventricular dissociation and junctional rhythm. One patient developed supraventricular tachycardia (SVT), which resolved after adenosine administration.

In the remaining patients, transient arrhythmias—including SVEs in three patients, SVT in two patients, atrioventricular dissociation in one patient, and accelerated junctional rhythm in one patient—resolved spontaneously following catheter manipulation and did not require pharmacological treatment.

However, intermittent third‐degree atrioventricular block developed in one patient after closure of a large ASD. Because the arrhythmia persisted despite corticosteroid therapy, permanent pacemaker implantation was required.

Another important complication observed in our study was thromboembolic events. One patient developed aphasia and right‐sided hemiparesis on the day after the procedure. Brain magnetic resonance imaging (MRI) demonstrated microembolic lesions in the branches of the left middle cerebral artery (MCA). The patient subsequently underwent a structured physical therapy program, and the neurological deficits gradually improved.

In another case, during transcatheter closure of an iatrogenic ASD in a patient who had previously undergone atrial septostomy during the neonatal period for critical pulmonary stenosis, embolization of an old organized thrombus originating from the pseudoseptum of the interatrial septum was detected simultaneously by TEE. Immediate thrombectomy was performed during the same session, and the patient was discharged without neurological sequelae.

Another relatively common complication was device migration. Device migration occurred in three patients after ASD closure. In all cases, the defect was successfully reclosed during the same session after retrieval of the migrated device using a snare catheter. A different device type was implanted in one patient, a larger device was selected in another patient, and in the third patient, the same migrated device was successfully repositioned.

### 3.2. Simultaneous Procedures and Balloon‐Assisted Techniques

Concomitant interventional procedures were performed during the same session as ASD closure in four patients. These included pulmonary balloon valvuloplasty in three patients and aortic balloon valvuloplasty in one patient (Table 1). In addition, balloon‐assisted techniques were employed for ASD closure in nine patients with large ASDs [7].

All patients underwent TTE and ECG evaluation before hospital discharge following the procedure. Device position was confirmed to be appropriate, with no evidence of residual shunting or atrioventricular valve dysfunction. Follow‐up outpatient evaluations were scheduled at 1, 3, and 6 months after the procedure. During follow‐up, all patients remained asymptomatic, and no residual shunt was detected.

### 3.3. Comparison of Measurement Techniques

In this study, several measurement strategies were compared with the final device waist diameter using Bland–Altman analysis to assess agreement and ICC to evaluate reliability. In Bland–Altman analysis, the LoA represent the range within which most differences between two measurement methods are expected to lie. When interpreting the results, both the magnitude of the mean bias and the width of the LoA were taken into consideration; techniques demonstrating smaller bias and narrower LoA were considered to show closer agreement with the reference measurement.

All measurement techniques demonstrated statistically significant correlations with the final device waist diameter (all *p* < 0.001).

Among the TEE methods, the cube root–derived diameter and the arithmetic mean both demonstrated high reproducibility, with ICC values of 0.940 (95% CI: 0.918–0.956) and 0.935 (95% CI: 0.911–0.953), respectively, and similarly favorable Bland–Altman profiles. The cube root–derived diameter showed minimal bias (−0.25 mm) and narrow LoA (−3.45 to +2.95 mm; Figure 2f), while the arithmetic mean demonstrated comparable results with a nearly identical bias (−0.22 mm) and only marginally wider LoA (−3.57 to +3.13 mm; Figure 2e). The marginal numerical difference between the two methods did not suggest a clinically meaningful distinction (Table 2).

**FIGURE 2:**
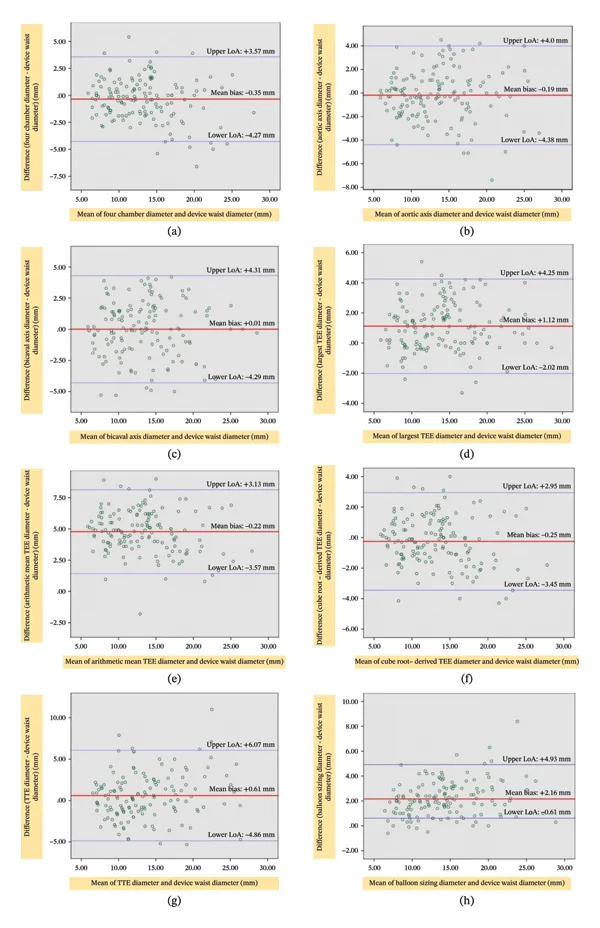
Bland–Altman plots demonstrating the agreement between defect diameter measurements obtained by different sizing methods (including transesophageal echocardiography, transthoracic echocardiography, and balloon sizing) and the final device waist diameter. (The *x*‐axis represents the mean of the measurement method and the device waist diameter, while the *y*‐axis represents the difference between the measurement method and the device waist diameter. The solid red line indicates the mean bias, and the thin blue lines represent the 95% limits of agreement [LoA]). (a): Comparison of defect diameters in the four chamber axis measured by TEE and device waist diameters (Bland–Altman graph). (b): Comparison of defect diameters in the aortic axis measured by TEE and device waist diameters (Bland–Altman graph). (c): Comparison of defect diameters in the bicaval axis measured by TEE and device waist diameters (Bland–Altman graph). (d): Comparison of the largest diameter of all axes measured by TEE and device waist diameters (Bland–Altman graph). (e): Comparison of the arithmetic mean of all axis defect diameters measured by TEE and device waist diameters (Bland–Altman graph). (f): Comparison of the cube root of the products of all axis defect diameters measured by TEE and device waist diameters (Bland–Altman graph). (g): Comparison of transthoracic defect diameters and device waist diameters (Bland–Altman graph). (h): Comparison of balloon sizing defect diameters and device waist diameters (Bland–Altman graph).

**TABLE 2:** Intraclass correlation coefficient (ICC).

Variables	Correspondence analysis	
	Intraclass correlation coefficient (95% CI)	*p*
TEE four‐chamber axis diameter	0.910 (0.877–0.934)	< 0.001
TEE bicaval axis diameter	0.900 (0.865–0.927)	< 0.001
TEE aortic axis diameter	0.905 (0.871–0.930)	< 0.001
^∗^Arithmetic mean of the TEE values	0.935 (0.911–0.953)	< 0.001
^∗^Cube root of TEE measurements	0.940 (0.918–0.956)	< 0.001
The largest defect diameter measured by TEE	0.925 (0.779–0.964)	< 0.001
TTE‐derived defect diameter	0.852 (0.798–0.891)	< 0.001
Balloon sizing diameter	0.881 (0.707–0.965)	< 0.001

*Note:* All defect measurement techniques were correlated with the final device waist diameter measurements.

^∗^Among all evaluated measurement techniques, both the cube root–derived diameter and the arithmetic mean of TEE measurements demonstrated the highest and similarly favorable levels of agreement with the final device waist diameter.

While the largest TEE‐derived diameter demonstrated excellent reproducibility (ICC = 0.925), Bland–Altman analysis revealed greater variability at the individual level, as reflected by wider and asymmetric LoA (mean bias: +1.12 mm; LoA: −2.02 to +4.25 mm). Individually analyzed axis‐specific TEE measurements demonstrated additional variability, with SDs ranging from 2.00 to 2.19 mm (Table 3).

**TABLE 3:** Bland–Altman analysis of defect diameter measurements compared with device waist diameter.

Measurement method	Bias (mean difference, mm)	SD (mm)	Lower LoA (mm)	Upper LoA (mm)
TEE four‐chamber axis diameter	−0.35	2.00	−4.27	+3.57
TEE aortic axis diameter	−0.19	2.14	−4.38	+4.0
TEE bicaval axis diameter	+0.01	2.19	−4.29	+4.31
The largest defect diameter measured by TEE	+1.12	1.60	−2.02	+4.25
^∗^Arithmetic mean of the TEE values	−0.22	1.71	−3.57	+3.13
^∗^Cube root of TEE measurements	−0.25	1.63	−3.45	+2.95
TTE‐derived defect diameter	+0.61	2.79	−4.86	+6.07
Balloon sizing diameter	+2.16	1.41	−0.61	+4.93

Abbreviations: LoA, limits of agreement; SD, standard deviation.

^∗^Overall, both the cube root–derived TEE measurement and the mean TEE diameter demonstrated close agreement with the final device waist diameter, showing minimal bias and relatively narrow limits of agreement.

In contrast, TTE and balloon sizing demonstrated lower concordance with the reference measurement, although correlations remained statistically significant for all techniques (Table 2, Figure 2). TTE exhibited the greatest variability among all evaluated methods (SD: 2.79 mm) and the widest LoA (−4.86 to +6.07 mm), whereas balloon sizing demonstrated a distinct profile with lower variability (SD: 1.41 mm) but a consistent positive bias (+2.16 mm), suggesting a systematic tendency toward overestimation (Figure 2h, Table 3).

Overall, both the cube root–derived diameter and the arithmetic mean of TEE measurements demonstrated favorable balances between precision and agreement for predicting the final device waist diameter, with no clinically meaningful difference between the two methods.

Bland–Altman plots comparing the final device waist diameter with the other measurement techniques are presented in Figures 2a–h.

Anatomical factors, including rim deficiency and defect morphology, are important determinants of device sizing and procedural success. In the present study, rim deficiency (defined as a rim length < 5 mm) was identified in 51 patients, with the anterior aortic rim representing the most commonly affected segment (*n* = 37).

Pearson’s chi‐square analysis demonstrated no statistically significant association between rim deficiency and procedural complications. Complication rates were comparable between the groups, occurring in 13.7% (7/51) of patients with rim deficiency and 13.3% (13/98) of those without rim deficiency (*χ* ^2^ = 0.006, *p* = 0.94). Furthermore, the odds ratio of 1.04 (95% CI: 0.39–2.80) indicated that rim deficiency was not associated with an increased risk of complications in this cohort.

### 3.4. Subgroup, Sensitivity, and Selection Bias Analyses

Within the predefined rim‐deficiency subgroup, the cube root–transformed and arithmetic mean TEE measurements maintained high reliability, demonstrating consistently high and similarly favorable correlations with the final device waist diameter (ICC: 0.932 and 0.933, respectively). Notably, the correlation coefficients for measurements obtained along the aortic axis were slightly lower in this subgroup compared with the overall cohort (ICC: 0.892 vs. 0.905). This modest reduction in precision may reflect the high prevalence of anterior aortic rim deficiency among these patients, which could complicate the acquisition of standardized axis‐specific measurements.

To assess potential selection bias, baseline demographic and echocardiographic characteristics of included and excluded eligible patients were compared. Age (6.88 ± 4.38 vs. 6.67 ± 4.45 years, *p* = 0.664), body weight (27.66 ± 19.55 vs. 25.65 ± 15.90 kg, *p* = 0.309), and TTE ASD diameter (14.05 ± 5.57 mm vs. 13.19 ± 4.11 mm, *p* = 0.114) were similar between the groups. Patients were excluded primarily because of incomplete archival data or the absence of simultaneous application of all imaging modalities during the procedure rather than differences in baseline clinical characteristics. These findings suggest that exclusion was driven primarily by the absence of complete multimodality imaging data rather than systematic anatomical differences between groups, thereby reducing the likelihood of anatomy‐related selection bias. Nevertheless, since procedural variables such as rim status and device type were unavailable for excluded patients, the possibility of residual selection bias cannot be entirely excluded.

As a sensitivity analysis, ICCs were recalculated separately for patients with deficient rims (*n* = 51) and those without deficient rims (*n* = 98). High agreement was observed across all measurement methods in both subgroups. In patients with deficient rims, ICC values ranged from 0.813 to 0.933, whereas in those without deficient rims, they ranged from 0.871 to 0.944. Notably, the arithmetic mean of TEE measurements and the cube root–derived diameter consistently demonstrated similarly high levels of agreement in both groups (ICC = 0.933 and 0.932 in patients with deficient rims; ICC = 0.937 and 0.944 in patients without deficient rims, respectively). In contrast, TTE‐derived defect diameter showed comparatively lower ICC values in both subgroups (0.813 and 0.871, respectively), though agreement remained acceptable across all methods. Despite these differences in ranking, agreement remained excellent for all measurement methods, and the overall pattern of ICC estimates was highly similar regardless of the presence of a deficient rim.

Exploratory analyses were performed to evaluate the association between procedural complications and device oversizing, rim deficiency, and device type. Device oversizing, defined as the difference between implanted device size and the mean TEE‐derived defect diameter, did not differ significantly between patients with and without complications (3.50 ± 1.84 mm vs. 3.19 ± 2.02 mm, respectively; *p* = 0.523). Similarly, complication rates were comparable in patients with and without deficient rims (13.7% vs. 13.3%, respectively; *p* = 0.938), and no significant association was observed between device type and the occurrence of complications (*p* = 0.621). Given the small number of adverse events, these analyses were substantially underpowered, and the absence of statistical significance should not be interpreted as evidence of absent or negligible clinical effects.

## 4. Discussion

Advances in interventional cardiology have enabled transcatheter treatment of many structural heart diseases, including ostium secundum ASD, which was previously managed exclusively by surgical repair. Accurate defect sizing remains essential for appropriate device selection, traditionally relying on preprocedural TTE and intraprocedural balloon sizing under TEE guidance [4].

Despite technological advances, accurate ASD sizing remains challenging, especially in large defects, those with deficient rims, and multiple ASDs [8]. Precise morphological assessment and appropriate device selection are critical determinants of procedural success and long‐term outcomes. Although three‐dimensional (3D) TEE (3D‐TEE) offers superior evaluation of defect morphology and multiple defects compared with 2D‐TEE, its routine application may be limited in small children.

TEE‐guided transcatheter ASD closure is a safe and effective procedure. In addition to providing detailed assessment of ASD morphology and size for treatment planning, especially in complex or multiple defects, TEE is indispensable for intraprocedural guidance, accurate device deployment, and early identification of complications, including atrioventricular valve dysfunction and residual shunting [8].

However, TEE has several limitations, including the need for sedation or general anesthesia and prolonged procedural time [4]. Ackermann et al. [9] found that TEE‐guided ASD closure without fluoroscopy or balloon sizing was as safe and effective as fluoroscopy‐guided closure with balloon sizing. Although preprocedural TTE measurements were similar between groups, balloon sizing yielded significantly larger defect diameters. While this strategy may be suitable in selected patients, it may be less appropriate in those with large or multiple ASDs and floppy or deficient rims.

Balloon sizing, particularly with the stop‐flow technique, has traditionally been considered the gold standard for ASD sizing and remains valuable in patients with complex defect morphology or thin interatrial septa despite declining use in some centers. However, its major limitation is the tendency to overestimate defect size, potentially leading to oversized device selection and an increased risk of cardiac erosion [3, 7]. Measurement accuracy is influenced by septal thickness; while generally reliable in thick septa, thin and highly compliant septal tissue may predispose to overstretching and greater measurement variability. Additional disadvantages include distortion of defect morphology, prolonged procedure time, increased radiation exposure and cost, and transient bradycardia or hypotension related to impaired diastolic ventricular filling during prolonged balloon inflation [5].

Rao et al. [10] reported a strong correlation between balloon‐stretched ASD diameters and 2D‐TEE measurements, proposing the following formula: stretched diameter = 1.05 × TTE diameter + 5.49. However, this equation is primarily applicable to large defects and may result in device oversizing when applied to smaller ASDs.

In the present study, balloon sizing showed good agreement with the final device waist diameter, with a mean bias of +2.16 mm and 95% LoA ranging from −0.61 to +4.93 mm on Bland–Altman analysis (Figure 2h), indicating modest overestimation within clinically acceptable limits. Intraclass correlation analysis further demonstrated good, although not excellent, reproducibility, supporting the overall reliability of balloon sizing despite systematic measurement bias.

Taken together, these findings suggest that although balloon sizing provides useful adjunctive information for device selection, its tendency to overestimate defect size and wider LoA compared with composite TEE‐based measurements may limit its use as a standalone sizing method. Therefore, selection of slightly smaller devices may be appropriate in selected cases, and refined predictive models may further improve device selection.

Bartakian et al. [11] evaluated the use of TEE and TTE for transcatheter ASD closure in children and found no significant difference between balloon stop‐flow diameters in the TEE group and defect diameters measured by TTE, concluding that both modalities were equally effective for device size determination. However, their cohort included only patients with simple ASDs and excluded multiple defects, a limitation acknowledged by the authors.

In our study, TTE‐derived measurements showed the weakest agreement with the final device waist diameter among all sizing methods. Although intraclass correlation analysis demonstrated a statistically significant association, agreement was lower than that observed with composite TEE‐based measurements. Bland–Altman analysis further revealed a small positive mean bias (+0.61 mm) with relatively wide LoA (−4.86 to +6.07 mm), indicating substantial measurement variability (Figure 2g). These findings suggest that while TTE measurements may approximate the final device waist diameter at the population level, their precision for individual device selection is limited.

The observed variability likely reflects inherent limitations of TTE, including suboptimal acoustic windows, patient‐specific anatomical factors, and assessment based on limited imaging planes. Therefore, TTE should be considered a complementary rather than definitive sizing tool and interpreted alongside TEE findings and, when appropriate, intraprocedural sizing techniques (Table 2, Figure 2g).

Sah et al. [12] investigated a TTE‐based scaling formula for predicting optimal device size before transcatheter ASD closure. A scaled ASD diameter was calculated by multiplying the mean diameter obtained from three standard TTE views (parasternal short‐axis, apical four‐chamber, and subcostal bicaval) by a correction factor of 1.2. The predicted device size showed a strong correlation with the final implanted device size determined by conventional TEE assessment and stop‐flow balloon sizing, leading the authors to conclude that scaled TTE‐derived measurements were comparable to TEE‐guided sizing.

However, the findings of the present study differ from those of Sah et al. TTE‐derived measurements showed the weakest agreement with the final device waist diameter among all sizing methods evaluated (Table 2, Figure 2g). This discrepancy may be related to differences in study populations, imaging quality, operator experience, or the use of a fixed correction factor across diverse defect morphologies and sizes. Our findings suggest that although TTE provides useful preliminary information, it should be interpreted cautiously when used to guide device size selection.

Erdem et al. [13] compared TTE‐guided and TEE‐guided transcatheter ASD closure and found that both echocardiographic measurements and selected device sizes were significantly larger in the TEE‐guided group. The authors concluded that TTE guidance may be safe and effective in patients with adequate septal rims, favorable acoustic windows, and selected complex ASDs, whereas TEE guidance remains preferable for very large defects, floppy rims, multiple defects, or suboptimal acoustic windows. Gupta et al. [5] compared ASD sizing based exclusively on balloon sizing versus TEE and reported a higher rate of unsuccessful device closure in the balloon sizing group, suggesting that the additional anatomical information provided by TEE may improve procedural success. Similarly, Sadeghian et al. [14] demonstrated a strong correlation between TEE‐derived ASD diameters and balloon sizing measurements, while also showing that the discrepancy between the two methods decreased as TEE‐measured defect size increased.

Narimani et al. [15] compared 3D‐TEE with balloon sizing in adults undergoing ASD closure and found a strong correlation between the two methods. The ASD area measured by 3D‐TEE showed the highest correlation with balloon sizing in round defects, whereas the maximum 3D‐TEE diameter correlated best in oval defects. In addition, agreement between the two methods was stronger in patients without floppy septal rims [15].

In 2022, Vesal et al. [16] compared TTE, 2D‐TEE, and balloon sizing in 39 children undergoing transcatheter ASD closure. Similar to our findings, they reported a significant correlation between 2D‐TEE measurements and device size. They also demonstrated that the mean ASD diameter determined by balloon sizing was significantly larger than the diameters measured by either 2D‐TEE or TTE. Furthermore, ASD dimensions obtained by TTE and TEE correlated with device waist diameter and, when combined with simple predictive equations, provided useful guidance for device size selection in patients undergoing transcatheter ASD closure.

ASDs are not always circular with regular margins; rather, they are frequently oval and may exhibit different dimensions across multiple imaging planes. Consequently, several investigators have attempted to develop mathematical formulas incorporating measurements from multiple axes to improve device size prediction. Narimani et al. [15] classified ASD morphology as either round or oval using an arbitrary cutoff ratio of 1.3 between the maximum and minimum defect diameters. Similarly, Zanchetta et al. [17] proposed the equation $d=\sqrt{\left(a\times b\right)}$, where *a* and *b* represented the major defect diameters measured by intracardiac echocardiography in the aortic and four‐chamber views, respectively, and *d* corresponded to the selected device diameter. Consistent with these approaches, we calculated the geometric mean of the maximal defect diameters obtained from three TEE imaging planes as $\left[\sqrt[{3}]{\left(a\times b\times c\right)}\right]$ and compared this value with the final device waist diameter.

In this study, single‐plane TEE measurements obtained from standard imaging views demonstrated variable levels of agreement with the final device waist diameter. Among the evaluated methods, both the cube root–derived diameter and the arithmetic mean demonstrated high reproducibility with similarly high ICC values (cube root: ICC = 0.940, 95% CI: 0.918–0.956; arithmetic mean: ICC = 0.935, 95% CI: 0.911–0.953) (Table 2), and Bland–Altman analysis revealed no clinically meaningful difference between the two methods. Given its simplicity, the arithmetic mean may be preferred in routine clinical practice.

Exploratory analyses did not identify oversizing, deficient rim anatomy, or device type as predictors of procedural complications; however, these findings should be interpreted cautiously given the limited number of adverse events.

In patients with small ASDs and adequate acoustic windows, TTE may provide sufficient anatomical information for device selection. However, in the presence of large defects, multiple ASDs, deficient septal rims, or associated cardiac anomalies, TEE‐guided closure offers more comprehensive anatomical assessment and may reduce the risk of procedural complications, including device malposition or embolization. Notably, device migration occurred in three patients in our cohort, all of whom had multiple defects and inadequate septal rims. These observations support the use of TEE guidance and, when appropriate, adjunctive balloon sizing in anatomically complex cases to facilitate optimal device selection and potentially reduce complication rates.

Although the final device waist diameter cannot be considered a fully independent reference standard, it may reflect more than the operator’s initial sizing decision. Following deployment, the device waist adapts to the native defect anatomy and surrounding tissue characteristics, particularly in noncircular defects. Therefore, the final waist diameter may represent a clinically relevant measure of device–defect interaction. Nevertheless, because device selection and postdeployment waist dimensions are inherently related, our findings should be interpreted primarily as reflecting agreement with operator‐selected device sizing rather than true anatomical sizing accuracy.

In recent years, transcatheter ASD closure has increasingly been performed using echocardiographic guidance alone in selected patients [18]. However, in complex anatomical settings, combining multiple sizing techniques, particularly TEE‐based measurements, may improve concordance with the operator‐selected device size and support procedural planning [19]. Ensuring procedural safety remains paramount.

The present study included four different septal occluder devices from distinct manufacturers. Although Amplatzer Septal Occluder accounted for the majority of implantations (70.5%), different devices vary in nitinol wire density, fabric composition, and waist flexibility, which may theoretically influence postdeployment waist diameter and thereby affect agreement analyses. The predominance of a single device type likely limited the overall impact of device‐specific characteristics on our findings; however, formal device‐stratified analyses were not performed due to the small sample sizes in non‐Amplatzer groups.

## 5. Conclusion

Appropriate device selection during transcatheter ASD closure is essential for achieving complete defect occlusion while minimizing procedure‐related complications. Although numerous ASD sizing techniques have been investigated, no consensus has yet been established regarding the optimal method for device selection. In the present study, we compared multiple measurement techniques to identify the approach that demonstrated relatively favorable agreement characteristics with the final implanted device size.

ASDs are frequently noncircular and predominantly oval in morphology. Following transcatheter closure, deployment of the occlusion device often results in a more circular configuration of the defect. Consequently, measurements obtained from a single TEE imaging plane may not adequately represent the true 3D geometry of the defect and may therefore be insufficient when used alone for device size determination.

On this basis, we propose that composite TEE‐based measurements, such as the cube root of the product of multiple defect dimensions or the arithmetic mean of TEE‐derived diameters obtained from different imaging planes, may provide a more representative assessment of effective defect size and may facilitate more concordant device selection.

Precise device selection is critical during transcatheter ASD closure, as both oversizing and undersizing may increase the risk of procedure‐related complications. Although statistically significant differences were observed among the various measurement techniques evaluated, an overall agreement was maintained across methods. Importantly, composite approaches incorporating multiple TEE‐derived measurements demonstrated slightly closer agreement with the final device waist diameter compared with individual measurement techniques, highlighting their potential value in achieving closer agreement with operator‐selected device sizing, which may in turn support procedural outcomes.

Analysis of complication rates according to rim status revealed no significant difference between patients with and without rim deficiency. However, given that only 20 adverse events were recorded, these analyses were underpowered to detect clinically meaningful differences, and the absence of statistical significance should not be interpreted as evidence of an absent effect. Procedural outcomes are likely influenced by multiple anatomical and technical factors that could not be fully evaluated in this cohort. Despite anatomical complexity, appropriate device sizing remains a key factor in minimizing procedural risk.

Although device selection should ultimately be individualized for each patient, integrating multiple measurement techniques—particularly multiaxis TEE measurements and composite indices derived from these measurements—may improve concordance between preprocedural measurements and the operator‐selected device size, thereby supporting more consistent sizing decisions and potentially reducing procedure‐related complications.

### 5.1. Limitations

This study has several limitations. First, it was conducted as a single‐center study, which may limit the generalizability of our findings. Second, while 3D‐TEE has been shown in some studies to provide superior assessment of ASD diameter, planimetric area, and complex anatomy, this technology was not utilized in our cohort. Since 3D‐TEE is not yet universally available in all clinical settings, our results reflect current practices in centers relying on 2D‐TEE and traditional sizing methods.

In addition, interobserver variability and reproducibility between echocardiographers were not formally assessed. Because of the retrospective design, measurements were derived from routine clinical examinations rather than under a predefined reproducibility protocol. However, all echocardiographic measurements were routinely reviewed by multiple experienced operators during clinical evaluation, and the final recorded values reflected a consensus‐based assessment within the procedural team, thereby minimizing potential measurement discrepancies.

Importantly, given that the observed differences between the cube root–derived and arithmetic mean methods were small (bias difference < 0.03 mm), the absence of formal reproducibility testing limits the ability to determine whether these differences are clinically meaningful.

Although the final device waist diameter cannot be regarded as a definitive anatomical gold standard, it was used as a clinically relevant procedural reference because it reflects the outcome of comprehensive preprocedural and intraprocedural assessment. Because device selection is based on the integration of multiple anatomical and imaging parameters rather than any single measurement, some degree of circularity is unavoidable when implanted device size is used as the reference comparator. Given the absence of a universally accepted gold standard for ASD sizing and the inherent limitations of currently available sizing methods, this approach provides a pragmatic reference for evaluating the agreement between imaging modalities and device selection in routine clinical practice. Therefore, the findings should be interpreted as reflecting concordance with the final implanted device size rather than the true anatomical defect diameter.

The complication analyses in this study are additionally limited by the small number of adverse events (*n* = 20). The associations evaluated between procedural complications and rim deficiency, device oversizing, and device type should therefore be considered exploratory rather than definitive. These analyses were not powered to detect statistically meaningful differences, and the results should be interpreted as hypothesis‐generating. Adequately powered prospective studies are needed to further investigate the relationship between these anatomical and procedural variables and clinical outcomes.

Despite efforts to minimize selection bias, the exclusion of nearly half of the screened population due to incomplete multimodality imaging datasets remains a potential limitation. Importantly, TTE‐derived defect diameter measurements were available for the majority of excluded patients, and no statistically significant difference was observed between included and excluded patients in age, body weight, or TTE‐derived ASD diameter. This suggests that exclusion reflected a logistical and archival limitation rather than a systematic anatomical difference between groups. However, procedural and anatomical variables beyond defect diameter—such as rim status, defect morphology, and device type—were unavailable for excluded patients, and residual selection bias in these dimensions cannot be entirely excluded. Consequently, the generalizability of our findings should be interpreted with caution, particularly in centers where the simultaneous application of all imaging modalities is not routine practice.

Future prospective, multicenter studies with predefined imaging protocols are warranted to confirm these findings across more diverse patient populations.

Furthermore, the study spans a nearly ten‐year period (2016–2025), during which subtle evolution in operator experience, imaging technology, and procedural strategy may have occurred. Although all procedures were performed by the same experienced operators using standardized protocols at a single center and no formal changes in imaging equipment or device selection strategy were introduced during the study period, temporal effects cannot be entirely excluded. A formal analysis of potential time‐period effects was not performed, and this represents an additional limitation that prospective studies with predefined temporal checkpoints could address.

Additionally, the follow‐up duration of 6 months may be insufficient for detecting late‐onset device‐related complications, such as cardiac erosion, late arrhythmias, or thromboembolic events, which may manifest beyond this period. Longer‐term follow‐up studies are warranted to more comprehensively assess the safety profile of transcatheter ASD closure.

Furthermore, although four different occluder devices were used, the small number of non‐Amplatzer implantations precluded a formal subgroup analysis stratified by device type, and device‐specific waist characteristics as a potential source of variability cannot be entirely excluded.

## Funding

This study was supported by the Erciyes University Scientific Research Projects Coordination Unit (Project No.: TSG‐2019‐8336).

## Disclosure

The funders had no role in the study design, data collection, data analysis, decision to publish, or preparation of the manuscript.

## Conflicts of Interest

The authors declare no conflicts of interest.

## Data Availability

The data that support the findings of this study are available on request from the corresponding author. The data are not publicly available due to privacy or ethical restrictions.
